# A case of endovascular therapy for treating idiopathic arterial deteriorations of unknown etiology

**DOI:** 10.1016/j.ijscr.2020.09.178

**Published:** 2020-09-30

**Authors:** Sohei Matsuura, Toshio Takayama, Takashi Endo, Takafumi Akai, Toshihiko Isaji, Katsuyuki Hoshina

**Affiliations:** Division of Vascular Surgery, Department of Surgery, The University of Tokyo, Tokyo, Japan

**Keywords:** CT, computed tomography, EVT, endovascular therapy, FMD, fibromuscular dysplasia, RA, renal artery, SAM, segmental arterial mediolysis, SCARE, Surgical case report, vEDS, vascular Ehlers-Danlos syndrome, Artery rupture, Pseudoaneurysm, Endovascular therapy

## Abstract

**Introduction:**

Peripheral artery pseudoaneurysm as a consequence of arterial deterioration is relatively rare in young populations, who typically lack an atherosclerotic background. Such pseudoaneurysms are known to pose a risk of rupture, which is correlated with high mortality and morbidity rates. Pseudoaneurysms are more prone to rupture than true aneurysms are, as their vessel walls tear more easily. We present the case of a 50-year-old patient who had multiple arterial deteriorations.

**Case presentation:**

The patient experienced backache, and computed tomography revealed a tear of the right renal artery, and a pseudoaneurysm of the left internal iliac artery. He had a history of graft replacement for the right superficial artery pseudoaneurysm, which had been occluded. Although various tests were performed for differential diagnosis, the etiology was unidentified. Considering the suspected vascular fragility and failure of previous direct intervention, these 2 lesions were treated by placing endografts simultaneously.

**Discussion:**

We investigated various diseases causing vascular fragility in the reported case, such as vascular Behçet disease, vascular Ehlers-Danlos syndrome, fibromuscular dysplasia, and segmental arterial mediolysis. However, these were all excluded and the etiology remains unclear. Progress in endovascular techniques enables the use of minimally invasive treatment in patients with vascular fragility.

**Conclusion:**

When vascular fragility may exist, endovascular treatment is a desirable option, as it can be performed repetitively and is less invasive.

## Highlights

• A 50-year-old patient presented with multiple idiopathic arterial deteriorations.
• He had a renal artery tear and a pseudoaneurysm of the left internal iliac artery.
• Previous direct intervention failed and we suspected vascular fragility.
• The two lesions were treated by simultaneously placing endografts.
• Endovascular treatment is a desirable option in the case of vascular fragility.

## 1 Introduction

Peripheral artery pseudoaneurysm as a consequence of arterial deterioration is relatively rare in young populations lacking the typical atherosclerotic background and is known to have potential risk of rupture, which correlates with high mortality and morbidity rates [1,2]. Unlike a true aneurysm where the vessel wall maintains the typical three-layer anatomical structure [3], a pseudoaneurysm is more prone to rupture because the vessel wall tears more easily [4].

We present the case of a relatively young patient who developed two simultaneous pseudoaneurysms, one of which was caused by the spontaneous arterial tear, and one previous pseudoaneurysm of unknown pathology in different arteries.

The patient provided written informed consent for the publication of this case and accompanying images, and his anonymity was ensured. According to the rules of medical ethics in our institution, ethical review is not required for case reports. This work was reported in line with the SCARE criteria [5,6].

## 2 Case presentation

A 50-year-old man admitted to a nearby hospital for long-lasting back pain that had been worsening over the previous two months. His medical history was significant for deep vein thrombosis in the left lower extremity and a prior pseudoaneurysm repair in the right superficial femoral artery with a prosthetic graft, which later became occluded. He had also undergone an ileectomy for a small bowel obstruction caused by multiple ulcers in the terminal ileum. He had no remarkable family history of cerebrovascular or cardiovascular disease. He was a current smoker with a 60 pack-year smoking history.

**Fig. 1:**
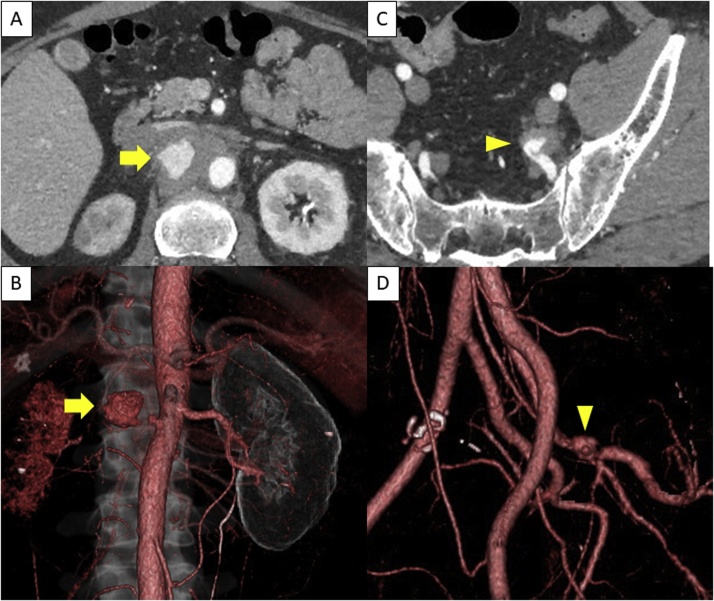
Preoperative computed tomography. (A) Blood leaked from the vessel and pooled at the right of the aorta (arrow) and (B) the right renal artery is not enhanced. The left internal iliac pseudoaneurysm (C) was also observed as a saccular aneurysm (D) (arrowhead).

Laboratory tests were significant for inflammation, mild renal dysfunction, and coagulopathy (fibrinogen level, 655 mg/dL; D-dimer level, 1.6 μg/mL). Contrast-enhanced CT revealed a 23 mm pseudoaneurysm in the right RA and a solitary 23 mm aneurysm in the left internal iliac artery (Fig. 1). He was transferred to and hospitalized at our institution on the day of the initial diagnosis.

Antihypertensive therapy and analgesics were administered immediately. Radiologists attempted coil embolization of the right RA on the third day after admission; however, the EVT was unsuccessful because the right RA was torn at the orifice, and the guide wire rapidly migrated into the extravascular space.

**Fig. 2:**
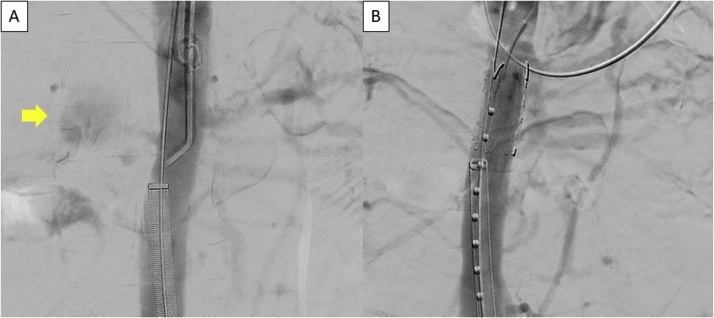
Intraoperative digital angiography of the right renal artery. Aortogram before the stent graft replacement shows the slightly enhanced origin of the right renal artery and blood pool to the right of aorta (arrow) (A). The right renal artery was excluded, and the pseudoaneurysm was no longer observed after the stent graft replacement (B).

**Fig. 3:**
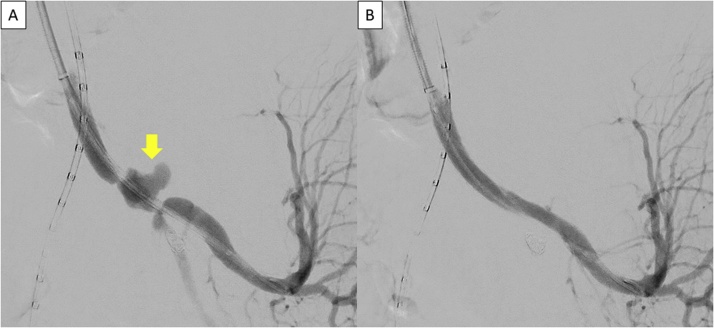
Intraoperative digital angiography of the left internal iliac artery. Angiography showing the left internal iliac pseudoaneurysm (arrow) before the stent graft replacement (A). The aneurysm was excluded, and no endoleak was observed after the stent graft replacement (B).

We performed EVT on the 10th day after admission. Direct puncture was performed to access the left common femoral artery, and the left axillary artery was accessed by surgical cutdown. Blood flow to the entire right RA was sacrificed to effectively isolate the pseudoaneurysm. The left RA was cannulated from the axillary access, and a 23 × 33 mm Excluder Aortic Extender (W.L. Gore & Associates, Flagstaff, AZ, USA) was deployed in the abdominal aorta to thoroughly cover the right RA (Fig. 2). The left RA was also partially covered. The left internal iliac aneurysm was situated at the bifurcation of the superior and inferior gluteal arteries, and the superior gluteal artery flow was spared to prevent postoperative gluteal claudication. The left inferior gluteal artery was cannulated and embolized through the axillary access, followed by cannulation of the left superior gluteal artery and advancement of a 7-Fr guiding sheath. A 6 × 59 mm Gore Viabahn VBX balloon-expandable endoprosthesis (W.L. Gore & Associates) was deployed to cover from the left internal iliac artery to the left superior gluteal artery (Fig. 3). The final angiography revealed no endoleak from either aneurysm. The operative time was 187 min. Of note, no vascular fragility was observed during exposure and handling of the arteries.

**Fig. 4:**
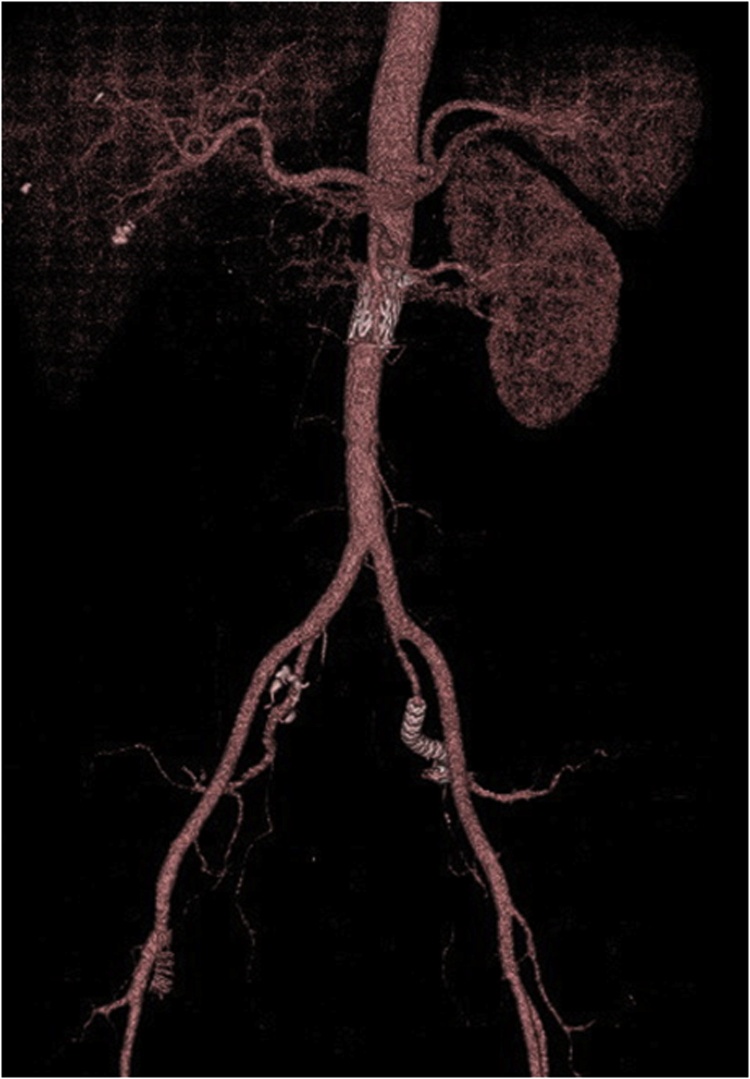
Postoperative 3D computed tomography. No endoleak was observed, and the right renal artery was not enhanced.

Although the patient was intubated for five days after surgery because of hypertensive heart failure secondary to renal hypertension, renal function gradually improved to baseline and the preoperative back pain resolved. Contrast-enhanced CT on the 12th day after surgery revealed no enhancement of either pseudoaneurysm (Fig. 4). The patient was discharged 16 days after surgery without gluteal claudication or other adverse events.

The patient was tested for HLA-A26/B51 and COL3A1 genetic abnormalities, and the results were unremarkable. Other than pseudoaneurysms, the patient exhibited no symptoms of Behçet disease, including uveitis and genital ulcers. Therefore, vascular Behçet disease and vEDS were excluded from the diagnosis. Specialists were consulted to analyze the possibility of collagen diseases, but no characteristic features in his face or habitus typical of other Marfan-like syndromes, such as Loeys-Dietz syndrome, were identified.

**Fig. 5:**
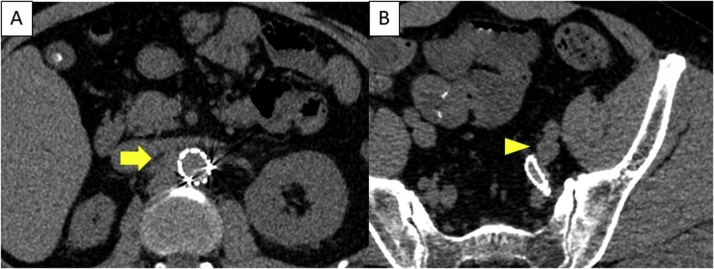
One-year surveillance computed tomography after surgery. (A) No blood leaked from the vessel at the right of the aorta (arrow). (B) The left internal iliac pseudoaneurysm decreased in the size (arrowhead).

Consequently, no extravasation from the right RA and no increase in the size of left iliac artery aneurysm has been noted in the last 12 months to date (Fig. 5).

## 3 Discussion

This report discusses a patient who has experienced three peripheral pseudoaneurysms in different arteries. There are many possible differential diagnoses for artery aneurysms. Primary causes include atherosclerosis; dissection; SAM; FMD; vasculitis; such as vascular Behçet disease; and vascular collagen diseases such as Marfan syndrome, vEDS and Loeys-Dietz syndrome. Secondary causes are trauma, infection, and inflammation. Most reported cases of pseudoaneurysms are secondary to trauma (including iatrogenic causes) or inflammation (e.g., pancreatitis) [2,7,8]. Although we investigated various diseases causing vascular fragility in the reported case, the etiology remains unclear.

Because pseudoaneurysms are nearly ruptured lesions, multiple artery ruptures or pseudoaneurysms are often related with vascular fragility, such as vEDS or vascular Behçet disease. Both these diseases are diagnosed exclusively with genetic testing, and the patient in this case did not have genetic mutations in either COL3A1 or HLA-B51/A26, despite presenting vascular symptoms consistent with either disease.

This patient was relatively young for atherosclerotic disease and had no cardiovascular risk factor aside from a smoking habit. These patient characteristics indicate that FMD should be considered as a possible diagnosis. FMD is a non-atherosclerotic arterial disease that exhibits characteristic imaging features of focal (stenotic) or multifocal (beaded) lesions in medium to small size arteries [[9], [10], [11]]. Smoking is considered a risk factor, and although stenotic disease is more prevalent, aneurysm, dissection, or artery tortuosity are also observed [9,11]. Although these features are consistent with this case, the presence of multiple pseudoaneurysms is atypical for FMD.

SAM is another possible diagnosis for multiple artery ruptures without atherosclerosis, but this case did not meet the imaging diagnostic criteria for SAM [12,13]. Pathological evaluation was not possible with the EVT. Furthermore, his lack of prior bone fracture made osteogenesis imperfecta unlikely.

Progress in EVT technique has made it possible to use minimally invasive treatment in patients with vascular fragility. In the present case which had the history of failed direct surgical intervention, EVT was an appropriate option. It enabled us to treat multiple lesions in different anatomical locations simultaneously without anastomosis or arterial dissection, which might cause local inflammation and accompanying tissue fragility [2]. Although there are anatomic limitations with EVT (indication for use, device size, vascular access to the lesion, etc.), it remains a good candidate for cases involving multiple arterial deteriorations.

## 4 Conclusion

We experienced a case of idiopathic multiple pseudoaneurysms, including spontaneous tear of the renal artery, presenting at a younger age and successfully treated these with EVT. In cases where vascular fragility may exist, EVT is a desirable option, as it may be performed repetitively and is less invasive than direct surgery.

## Declaration of Competing Interest

The authors report no declarations of interest.

## Funding

This research did not receive any specific grant from funding agencies in the public, commercial, or not-for-profit sectors.

## Ethical approval

According to the rules of medical ethics in our institution, ethical review is not required for case reports.

## Consent

The patient provided written informed consent for the publication of this case and accompanying images, and his anonymity was ensured.

## Author contribution

SM, TT and KH conceived the case presentation. SM drafted the manuscript. TE, TT, TA, TI, and KH treated the patient. All authors read and approved the final manuscript.

## Registration of research studies

N/A.

## Guarantor

SM and TT are the guarantors and fully responsible for this work.
